# Mr. Bond's Case of Re-Inoculation

**Published:** 1805-01-01

**Authors:** J. Bond

**Affiliations:** Glastonbury


### *To the Editors of the Medical and Physical Journal.*

GENTLEMEN,

I Beg leave to communicate, through the medium of your useful Journal, the following fact relative to the vaccine disease, which seems to throw additional light on this very important improvement in the medical art; and, as far as it goes, to solve the question, whether the small-pox does ever supervene the cow-pox.

Eliza Comer, now aged two years, was inoculated on August 13, 1803, for the vaccine disease, in both arms, with virus procured from the Vaccine Pock Institution, and went through the disease in a perfectly favourable manner, without any pustules except those of the arms; the punctures on both of which rose with large areola around them: this is necessary to premise, in order to elucidate the following case. The small-pox becoming frequent in this town, the mother of the child desired to have her inoculated for it, as she was afraid, from what she had heard, that the cow-pox was not a preventive. Accordingly, to satisfy her, I inserted a portion of variolous matter in the *left arm only*, on the 1st of June last. On calling to see the child, four days after, I saw every appearance of the puncture rising; but expected that it would have died away again, as I have witnessed in other cases of re-inoculation; and my surprize was considerable, on visiting her the sixth day, to find the arm still rising with an efflorescence around it, with symptoms of fever and absorption. On the eighth day, a rash, resembling that which so often precedes the eruption of the small-pox, appeared, covering the greater part of the body, attended with a considerable degree of swelling and fever; the puncture on the arm still rising, and appearing more inflamed and elevated. Under these circumstances, being considerably considerably alarmed, expecting that the child was about to have the small-pox, and she being costive, I administered a dose of kali tart. and manna; the next morning the mother informed me, that the small-pox was coming out very thick. On seeing the child, I found the face and neck covered with at least an hundred pimples. On examining the arm, to see the state the puncture was in, I perceived that it was sinking, and evidently dying away; and the pustule above it, about half an inch, where she had been previously vaccinated, elevated, with an areola appearing round it. This led me to examine the state of the other arm; and with great satisfaction I perceived that the original puncture there was also risen, with a most beautiful areola around it, exactly similar in its appearance and dimensions to that which had taken place at the time of the original vaccination. The child being again costive, I repeated the kali tart. and manna; and by the thirteenth day I had the pleasure of seeing the appearances on the arms subside, the eruption vanish, and the child recover her former healthy state.

The inferences which I draw from the foregoing statement are: First, that the vaccine virus (or principle) lay dormant in this child's system. This is to be accounted for by the re-appearance of the original vaccine inflammation and areola around the former punctures on the arms; and the eruption, as well as I could distinguish, resembling more those of the vaccine than the variolous disease. Second, that the introduction of variolous fluid under the cutis of the left arm, had the effect of throwing the dormant vaccine principle into sufficient action to repel its admission into the system. And, third, may not the cases lately related of small-pox taking place after vaccination, be referred to the same laws? as it is not unlikely, that the same phenomena took place; for I should have probably considered this case as incomplete small-pox, had I not perceived the state of the original punctures on the arms. And if, on future observation, other practitioners should meet with similar cases, a wide field for inquiry and investigation will be opened; for, I am inclined to think that we are not yet properly acquainted with the pathology of the disease; and it may not be improper to conjecture, if it should be proved in other cases to remain dormant in the system, that the vaccine principle may prevent other contagious eruptive diseases: and I mean, in consequence, to keep a watchful eye over those

 that that I have vaccinated, to note what diseases they are obnoxious to, and what they escape.

I am, &c.

*Glastonbury, Nov.* 11, 1804. J. BOND.

P. S. I inoculated two other children for the small-pox on the same day, with some of the same matter, who had been previously inoculated for the vaccine disease, on the same day with Eliza Comer, with some of the same fluid that she was infected with; but in them I could not perceive the least effect. This I think proper to mention, to show that the vaccine fluid made use of, was in a proper state at the time of their vaccination: and also, that in some peculiar constitutions, that are probably originally more susceptible of the variolous disease, it makes more vigorous efforts to enter the system, than in those who are not so susceptible of it; and therefore, that in all, after the introduction of the vaccine fluid, the principle may remain dormant, though not obliged to exert or shew itself.

